# School belonging mediates the association between negative school climate and depressive symptoms among Chinese adolescents: a national population-based longitudinal study

**DOI:** 10.3389/fpsyg.2024.1368451

**Published:** 2024-05-24

**Authors:** Yongtian Yin, Qingxiang Su, Shaojie Li

**Affiliations:** ^1^Faculty of Education, Shandong Normal University, Jinan, China; ^2^Department of Undergraduate Academic Affairs, Shandong University of Traditional Chinese Medicine, Jinan, China; ^3^Jinan Yuying Middle School, Jinan, China; ^4^School of Public Health, Peking University, Beijing, China

**Keywords:** school belonging, negative school climate, depressive symptoms, adolescents, longitudinal study

## Abstract

**Background:**

A negative school climate is an important factor affecting students’ mental health. However, few studies have focused on the mechanisms underlying the relationship. This study aimed to explore the mediating effect of school belonging on the association between negative school climate and depressive symptoms among Chinese adolescents, using a nationwide longitudinal survey.

**Methods:**

We conducted a longitudinal study using data from the 2013 (T1) and 2014 (T2) waves of the China Education Panel Survey (CEPS). A negative school climate was assessed by school administrators’ reports. School belonging and depressive symptoms were evaluated using adolescents’ self-reports. We used a cross-lagged panel model to explore the mediating effect of school belonging on the association between negative school climate and depressive symptoms, adjusting for a set of covariates.

**Results:**

In total, 7,049 Chinese adolescents with a mean age of 12.9 years were included in this study. The results of the cross-lagged model showed that negative school climate at T1 was significantly negatively associated with school belonging at T2 (*β* = −0.089, 95%CI = −0.111–−0.067, *p* < 0.001), and was positively associated with depressive symptoms at T2 (*β* = 0.032, 95%CI = 0.012–0.054, *p* = 0.002). In addition, school belonging at T1 was significantly negatively associated with depressive symptoms at T2 (*β* = −0.025, 95%CI = −0.050–−0.001, *p* = 0.045). Mediation analysis showed that school belonging played a mediating role in the association between negative school climate and depressive symptoms (*β* = 0.002, 95%CI = 0.001–0.005, *p* = 0.041).

**Conclusion:**

Among Chinese adolescents, a negative school climate is associated with a greater risk of depressive symptoms. Improving school belonging may be helpful in decreasing the impact of a negative school climate on depressive symptoms in adolescents.

## 1 Introduction

Adolescence is an important period for mental growth and physical development. Physical and mental growth and expanded contact with society may render adolescents vulnerable to mental health problems (WHO, 2021). Depressive symptoms are the most common mental health problem in adolescents and constitute a major public health concern worldwide (Patel et al., 2007). A previous meta-analysis of 72 studies conducted over the past 20 years found that 34% of adolescents aged 10–19 years worldwide had depressive symptoms (Shorey et al., 2022). Studies have confirmed that depressive symptoms in adolescents not only affect their interpersonal interactions (Lee et al., 2021; Zheng et al., 2023) and growth (Akaltun et al., 2018) but also increase the incidence of suicide and self-harm (Pozuelo et al., 2022). Given the high prevalence and health risks of depressive symptoms in adolescents, governments and society should pay more attention to depression in adolescents.

The elucidation of early predictors of depressive symptoms and the design of interventions based on them are fundamental to the prevention and management of depressive symptoms in adolescents. Several previous meta-analyses have examined factors associated with depressive symptoms in adolescents, such as neighborhood risk, social media use, poor family relationships, negative life events, academic stress, abuse, and bullying (Stirling et al., 2015; Ivie et al., 2020; Tang et al., 2020). These factors can be divided into three categories: the individual, the family, and the school. The vast majority of adolescents spend most of their time in school, where they are required to complete their upper elementary and middle school semesters. Therefore, focusing on school factors related to depressive symptoms and enabling school administrators and teachers to implement targeted interventions may be a viable strategy for reducing depressive symptoms in adolescents.

School climate is regarded as an important factor in the academic performance and health of students, conceptualized as the school members’ perceived quality and character of their school environment (Wang and Degol, 2016). A previous U.S. longitudinal study found that a positive school climate was associated with fewer depressive symptoms among adolescents (Wong et al., 2021). Additionally, a study of high school students in China yielded the same results, namely, that high school students who perceived a more positive school climate reported fewer depressive symptoms than those who perceived a negative school climate (Nie et al., 2020). However, it is important to note that most studies assessed students’ perception of the school climate, which is highly heterogeneous and does not take into account the perspectives of administrators. Meanwhile, due to the different values and personal experiences of each student, their perceptions of school climate vary widely and there may be common method biases in exploring the association between school climate and students’ mental health if the school climate is evaluated only from the perspective of the students. Considering the capacity of school administrators to offer a more comprehensive and objective insight into the school, along with their potentially broader understanding of the overall school climate, employing evaluations by school administrators as a method to assess school climate could present a more comprehensive approach. Therefore, focusing on school climate as perceived by school administrators in relation to adolescent mental health may be more informative in developing future school-level intervention strategies, as.

According to bioecology, a school’s environment, conditions, and structure are distal factors that influence student development, having indirect effects through proximal factors such as student–student and student–teacher interpersonal relationships (Way et al., 2007; Wang and Degol, 2016). An integrated theory of school environment influences on health also suggests that the school environment can impact students’ cognition (practical reasoning abilities, cognitions and affiliations) and “drive” more or less healthy behaviors, thereby affecting their health (Bonell et al., 2013). Systems view of school climate posits that school climate directly impact idea of relationships within the school members (connection, support, affiliation, and belongingness) which in turn act on the patterns of behavior, decision-making and participation of the different role groups (Rudasill et al., 2018). Building on these frameworks, it is hypothesized that school climate may indirectly affect students’ mental health through their sense of belonging. However, no studies have focused on this possible mechanism of association. “School belonging” refers to a subjective feeling of acceptance, respect, and support from other school members, reflecting an emotional connection to the school (Goodenow, 1993). A previous cross-sectional study suggested that school belonging mediates the relationship between school climate and problematic Internet use (Zhai et al., 2020). Considering that problematic Internet use is strongly associated with depressive symptoms (Gámez-Guadix, 2014), this finding served as a guide for our study. However, it should be noted that because the previous study used a cross-sectional design, the directional relationships could not be determined. Therefore, to fill this gap in the literature, this study used a national longitudinal survey of adolescents to explore the longitudinal association between school climate, as rated by school administrators, and adolescents’ sense of school belonging and their depressive symptoms, as well as the mediating role of school belonging.

## 2 Methods

### 2.1 Participants

**Figure 1:**
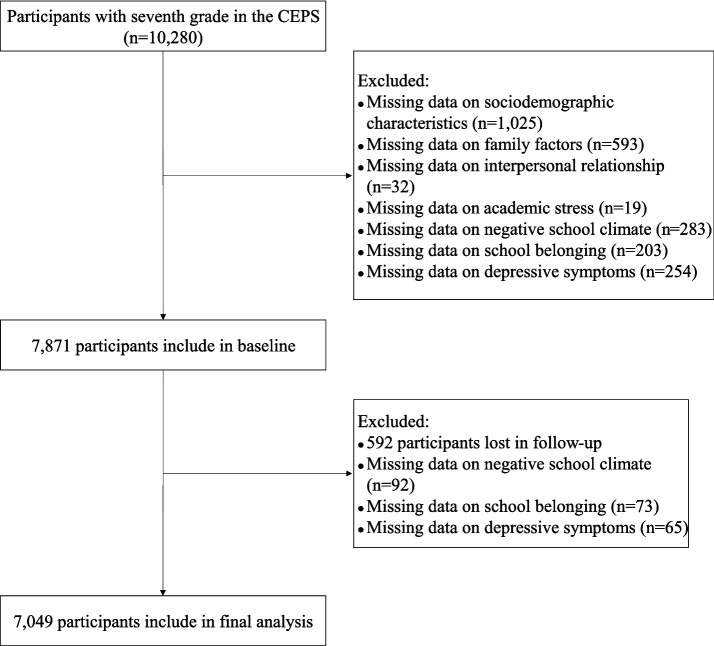
The sample selection process.

We conducted a longitudinal study using data from the 2013 (T1) and 2014 (T2) waves of the China Education Panel Survey (CEPS). The CEPS is a nationally representative survey of students, parents, teachers, and school administrators designed and implemented by the Renmin University of China (Jiang and Yang, 2023). A total of 112 schools within 28 districts and counties were randomly selected nationwide for the CEPS using a stratified, multi-stage, probability proportional to size sampling method. For school administrators, the CEPS project team initially contacted local districts/counties’ education bureau staff, who assisted in contacting the principals of selected schools. With the principals’ assistance, one school administrator was recruited from each school for the survey. The characteristics of school administrators are shown in Supplementary Table S1. In the 2013 wave, the CEPS recruited 10,280 seventh-grade students, conducting follow-up surveys on them in 2014. In this study, we first excluded samples with missing data on sociodemographic characteristics, family factors, health status, interpersonal relationships, academic stress, and negative school climate; 7,797 participants were included at baseline. Second, we excluded samples that were lost during follow-up or with missing data on depressive symptoms and school belonging. Ultimately, 7,049 participants were included in the study and the exact retention rate of the sample was 90.4%. Figure 1 shows the sample selection process. The Ethics Committee of Renmin University of China approved the implementation of the CEPS, and all participants (students, parents, teachers, and school administrators) signed informed consent forms.

### 2.2 Measurements

#### 2.2.1 Negative school climate

Negative school climate was assessed by school administrators’ reports at T1 and T2, which has been widely used in Chinese adolescent studies and has good internal consistency reliability and structural validity obtained through factor analysis (Lei and Yousheng, 2019; Guwen, 2023). School administrators were asked about the frequency of eight incidents at their school in the preceding week, including student performance and teacher performance. Student performance included: (1) student fights and brawls, (2) student vandalism, (3) student smoking, (4) student drinking, (5) gang activity on or off campus, (6) poor classroom discipline. Teacher performance included: (1) teachers’ scolding of students, and (2) teachers’ corporal punishment of students. Each event was scored using four categories: *never* (1 point), *one to four times* (2 points), *five to ten times* (3 points), or *more than ten times* (4 points). The total score was the sum of the eight event scores, ranging from 8 to 40 points. A higher score indicated a more negative school climate. This measure of negative school climate demonstrated good reliability in the current study, with Cronbach’s α = 0.810 at T1 and 0.728 at T2.

#### 2.2.2 School belonging

According to a previous review, school identification, student engagement, and attachment to school are important manifestations of school belonging (Slaten et al., 2016). Meanwhile, reference to the measurement of school belonging in previous CEPS-based studies (Li and Jiang, 2018; Jie and Lei, 2022), we used three items to evaluate the school belonging, which was adolescents’ self-reports at T1 and T2. Participants were asked to rate their recognition of four types of school life: (1) “My class has a good class culture,” (2) “I often participate in school or class-organized activities,” (3) “I feel close to the people at this school.” Each item was scored using a four-category scale: *fully disagree* (1 point), *relatively disagree* (2 points), *relatively agree* (3 points), and *fully agree* (4 points). The sum of all the items was the total score, with a higher score representing a higher sense of school belonging. This measure demonstrated good reliability in the current study, with Cronbach’s α = 0.721 at T1 and 0.723 at T2.

#### 2.2.3 Depressive symptoms

Depressive symptoms were assessed using a brief version of the Center for Epidemiologic Studies Depression Scale (CES-D) (Radloff, 1977). The scale consists of five items: (1) depression, (2) being too depressed to concentrate on doing things, (3) unhappiness, (4) finding life meaningless, and (5) sadness and grief. The participants were asked to rate the frequency of experiencing the five psychological symptoms in the preceding week. The response choices were *never* (1 point), *rarely* (2 points), *sometimes* (3 points), *often* (4 points), and *always* (5 points). The five items were summed to obtain a total score, with a higher score indicating a higher frequency of depressive symptoms. The scale demonstrated good reliability in the current study, with Cronbach’s α = 0.849 at T1 and 0.907 at T2.

#### 2.2.4 Covariates

Based on a previous systematic review and meta-analysis of psychosocial risk factors associated with depressive symptoms in adolescents in secondary schools in mainland China (Tang et al., 2020), we identified four aspects of covariates: sociodemographic characteristics, family factors, interpersonal relationships, and academic stress. The sociodemographic characteristics included age, sex, residence (urban or rural), and the presence of siblings (no or yes). The family factors included family economic level (low, medium, or high), father’s education level (below high school below, or high school and above), mother’s education level (below high school below, or high school and above), and family atmosphere. Family atmosphere was evaluated using the responses to three statements: (1) “My father often gets drunk” (yes = 0, no = 1), (2) “My parents often fight” (yes = 0, no = 1), and (3) “My parents have a good relationship with each other” (no = 0, yes = 1). The scores of the responses were summed to obtain the total score for family atmosphere, with a higher score indicating a better family atmosphere. Interpersonal relationships included number of friends and parents. Parentage was assessed using two items: closeness to the father (not close = 1, fairly close = 2, very close = 3) and to the mother (not close = 1, fairly close = 2, very close = 3). The sum of the two items comprised the total score for parentage, ranging from 2 to 6 points. Academic stress was reflected through perceived stress in three core subjects: Chinese, Mathematics, and English. Participants were asked to indicate how stressed they were about learning in the three subjects, with answers including four options: *very little* (1 point), *a little* (2 points), *somewhat* (3 points), and *very much* (4 points). The stress scores for the three subjects were added to obtain the total academic stress score, with a higher score indicating higher academic stress.

### 2.3 Statistical analysis

We conducted descriptive and correlation analyses using STATA 17.0 (Stata Corp, College Station, TX, United States). For continuous variables (e.g., negative school climate, school belonging), we calculated mean and standard deviation, while for categorical variables (e.g., sex, BMI), we calculated frequency (percentage). Pearson’s correlation analysis explored the relationships among negative school climate, school belonging, and depressive symptoms at both T1 and T2. Subsequently, we conducted constructed cross-lagged models using IBM SPSS AMOS 25.0 with robust maximum likelihood estimation. A 3 × 3 cross-lagged model was built, adjusting for all covariates, to analyze directional relationships among negative school climate, school belonging, and depressive symptoms at T1 and T2. Fit indices and cutoff value (Kline, 2023) for the cross-lagged model included: comparative fit index (CFI) and Tucker Lewis index (TLI) > 0.900, root mean square error of approximation (RMSEA) < 0.080, and the standard root–mean–square (SRMR) < 0.080. Referring to prior research on mediation analysis in longitudinal studies (Cole and Maxwell, 2003), we calculated the product of regression coefficients from T1 negative school climate to T2 school belonging, and from T1 school belonging to T2 depressive symptoms, representing the mediating effect of school belonging between negative school climate and depressive symptoms. We estimated 95% bootstrap confidence intervals (CI) for all paths and the aforementioned product using 5,000 bootstrap iterations. Statistical significance was determined by 95% CI not including 0 or *p* < 0.05.

## 3 Results

### 3.1 Descriptive statistics

In total, 7,049 Chinese adolescents with a mean age of 12.9 ± 0.9 years were included in the study. The mean scores for negative school climate, school belonging, and depressive symptoms at T1 and T2 were 9.9 ± 2.5, 9.7 ± 2.1, 9.0 ± 2.3, 8.9 ± 2.2, 10.0 ± 3.9, and 10.8 ± 4.6, respectively. Additional sample characteristics of adolescents are shown in Table 1.

**Table 1:** Descriptive analysis of participant characteristics.

Variables	
Age, mean ± SD	12.9 ± 0.9
Sex, *n* (%)	
Male	3,607 (51.2)
Female	3,442 (48.8)
Residence, *n* (%)	
Urban	3,433 (48.7)
Rural	3,616 (51.3)
Presence of siblings, *n* (%)	
Yes	3,875 (55.0)
No	3,174 (45.0)
Family economic level, *n* (%)	
Low	757 (10.7)
Medium	5,287 (75.0)
High	1,005 (14.3)
Father’s education, *n* (%)	
High school below	4,285 (60.8)
High school and above	2,764 (39.2)
Mother’s education, *n* (%)	
High school below	4,766 (67.6)
High school and above	2,283 (32.4)
Family atmosphere, mean ± SD	2.6 ± 0.7
The number of friends, mean ± SD	6.8 ± 3.1
Parentage, mean ± SD	5.4 ± 0.9
Academic stress, mean ± SD	7.2 ± 2.0
T1 Negative school climate, mean ± SD	9.9 ± 2.5
T2 Negative school climate, mean ± SD	9.7 ± 2.1
T1 School belonging, mean ± SD	9.0 ± 2.3
T2 School belonging, mean ± SD	8.9 ± 2.2
T1 Depressive symptoms, mean ± SD	10.0 ± 3.9
T2 Depressive symptoms, mean ± SD	10.8 ± 4.6

SD, standard deviation.

### 3.2 Pearson’s correlation analysis

Pearson’s correlation analysis (Table 2) showed that negative school climate at T1 was significantly correlated with school belonging at T2 (*r* = −0.138, *p* < 0.001) and depressive symptoms at T2 (*r* = 0.066, *p* < 0.001). Additionally, school belonging at T1 was negatively correlated with depressive symptoms at T2 (*r* = −0.154, *p* < 0.001).

**Table 2:** Correlation analysis.

Variables	1	2	3	4	5	6
1. Negative school climate-T1	1.000					
2. Negative school climate-T2	0.242	1.000				
*p* value	<0.001					
3. School belonging-T1	−0.098	−0.066	1.000			
*p* value	<0.001	<0.001				
4. School belonging-T2	−0.138	−0.072	0.417	1.000		
*p* value	<0.001	<0.001	<0.001			
5. Depressive symptoms-T1	0.051	0.036	−0.249	−0.206	1.000	
*p* value	<0.001	0.003	<0.001	<0.001		
6. Depressive symptoms-T2	0.066	0.037	−0.154	−0.236	0.419	1.000
*p* value	<0.001	0.002	<0.001	<0.001	<0.001	

### 3.3 Cross-lagged model

**Figure 2:**
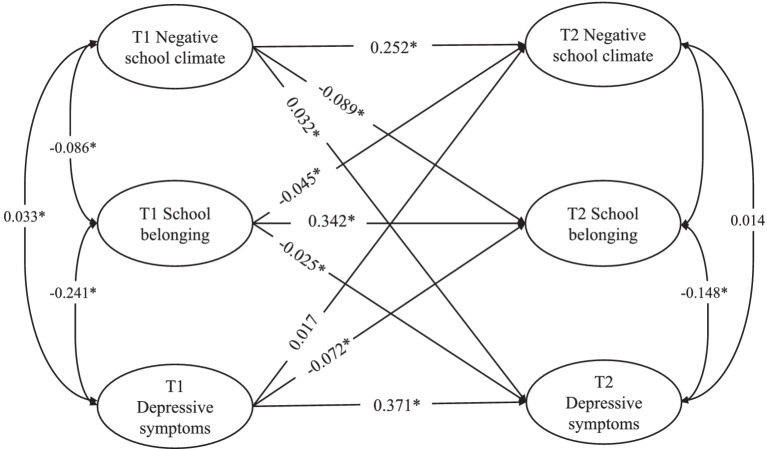
The cross-lagged model of negative school climate, school belonging and depressive symptoms at T1 and T2. ^*^p < 0.05; all coefficients are standardized coefficients.

The model fit index of cross-lagged model was the CFI = 0.994, the TLI = 0.971, the RMSEA = 0.021, and the SRMR = 0.014. Consistent with the correlation analysis results, the cross-lagged model (Table 3 and Figure 2) showed that negative school climate at T1 was significantly negatively associated with school belonging at T2 (*β* = −0.089, 95%CI = -0.111–-0.067, *p* < 0.001), and was positively associated with depressive symptoms at T2 (*β* = 0.032, 95%CI = 0.012–0.054, *p* = 0.002). In addition, school belonging at T1 was significantly negatively associated with depressive symptoms at T2 (*β* = −0.025, 95%CI = -0.050–-0.001, *p* = 0.045). Mediation analysis showed that school belonging played a mediating role in the association between negative school climate and depressive symptoms (*β* = 0.002, 95%CI = 0.001–0.005, *p* = 0.041).

**Table 3:** Bootstrapped estimation of each path of the cross-lagged model.

Path	*β*	SE	95% CI	*p*-value
Negative school climate T1 to Negative school climate T2	0.252	0.011	0.229 ~ 0.274	0.001
School belonging T1 to School belonging T2	0.342	0.013	0.318 ~ 0.367	<0.001
Depressive symptoms T1 to Depressive symptoms T2	0.371	0.014	0.343 ~ 0.397	0.001
Negative school climate T1 to School belonging T2	−0.089	0.011	−0.111 ~ −0.067	<0.001
Negative school climate T1 to Depressive symptoms T2	0.032	0.011	0.012 ~ 0.054	0.002
School belonging T1 to Negative school climate T2	−0.045	0.012	−0.069 ~ −0.021	<0.001
School belonging T1 to Depressive symptoms T2	−0.025	0.012	−0.050 ~ −0.001	0.045
Depressive symptoms T1 to Negative school climate T2	0.017	0.013	−0.010 ~ 0.042	0.200
Depressive symptoms T1 to School belonging T2	−0.072	0.013	−0.096 ~ −0.047	0.001

β, standardized regression coefficient; SE, standardized error; CI, confidence interval (bias-corrected bootstrap confidence intervals).

## 4 Discussion

In this study, we used national data in a longitudinal research design to explore the relationship between a negative school climate, school belonging, and depressive symptoms, as well as the mediating effect of school belonging among Chinese adolescents. Our study found that a negative school climate was significantly associated with school belonging and depressive symptoms. Further, school belonging mediated the relationship between negative school climate and depressive symptoms. This is the first study to explore these associations and mechanisms, providing scientific evidence for future interventions for adolescent depressive symptoms at the school level.

First, in the correlation analysis, the zero-order correlations indicated negative school climate at T1 was significantly and positively associated with depressive symptoms at T2, which is consistent with a previous longitudinal study in the United States (Wong et al., 2021). A longitudinal study of high school students in China also found similar results (Nie et al., 2020); however, because only one high school was selected as the sampling unit, the findings were less extrapolatable. This study extrapolated the association between school climate and depressive symptoms to the entire population of Chinese adolescents based on nationally representative data. Additionally, it is worth noting that this study used school climate as evaluated by school administrators, rather than as self-reported by students. Compared to students, school administrators may have a better understanding of school realities, including school systems, discipline, and culture (Smith and Andrews, 1989), than students do. Meanwhile, school climate, as reflected by school administrators, may make it easier to implement targeted interventions because the administrators themselves are the decision makers responsible for the day-to-day operation of the school. In this study, we evaluated negative school climate using student violence and delinquent behavior, classroom discipline, and corporal punishment by teachers. A higher score on negative school climate means that a school is likely to be more disorganized, resulting in students feeling less safe (Welsh, 2000), which, in turn, induces negative emotions such as depressive symptoms. This study also suggests that school administrators should actively establish systems and procedures to reduce a negative school climate, such as security patrols and student feedback channels, and enhance teacher education, which may help reduce the likelihood of students’ mental health problems.

Another important finding of this study was that school belonging mediated the association between a negative school climate and depressive symptoms. In a previous study that also explored the mechanism underlying the association between school climate and depressive symptoms, individual positive psychological qualities mediated the association (Nie et al., 2020). The mediating mechanism found in this study has two aspects: (1) the negative association between a negative school climate and school belonging, and (2) the negative association between school belonging and depressive symptoms. A previous cross-sectional study in Scotland found that an inclusive school climate was significantly and positively associated with higher school belonging (Long et al., 2021). A U.S. study of middle school students also found that four dimensions of students’ perceived school climate—safety, relationships, teaching and learning, and school environment—predicted varying degrees of school belonging (Renick and Reich, 2021). These studies support our finding of an association between a negative school climate and school belonging. Furthermore, the association between school belonging and depressive symptoms has been confirmed in previous studies. For example, a longitudinal study of LGBTQ adolescents found that school belonging was significantly and negatively associated with depressive symptoms and that school belonging mediated the association between peer victimization and depressive symptoms (Hatchel et al., 2018). An Australian study also found that school belonging was a significant predictor of depressive symptoms among adolescents (Parr et al., 2020). The interpersonal theory of suicide suggests that the interpersonal environment influences individuals’ depressive symptoms and that the interpersonal environmental indicator of frustrated belonging is one of the core factors contributing to suicide (Joiner, 2010; Van Orden et al., 2010).

Possible explanations for the above mediating role are the following. First, on the basis of social identity theory (Hogg, 2016) and self-categorization theory (Hornsey, 2008), students are important members of a school, and their social identity and sense of belonging to their school is an important expression of school connectedness (Klik et al., 2023). A negative school climate may make it difficult for students to integrate into school life, and they may be less motivated to participate in school activities because of a fear of being exposed to violence, thus reducing their sense of school belonging and increasing the risk of depressive symptoms. Second, middle school is an important life transition period for Chinese adolescents because they are required to study hard to enter college or a university. During this period, they spend far more time in school than at home, and school is an important interpersonal environment for them (Jia et al., 2009). A negative school climate can lead to heightened mistrust among adolescents towards school members, consequently affecting their interpersonal interactions. This can result in a perception of not being accepted or understood within the school environment. These circumstances can precipitate a loss of adolescents’ school belonging, leading to feelings of isolation and helplessness, which in turn may induce depressive symptoms as the absence of school support systems and emotional connections.

Our study has a potential value for the development of prevention and intervention strategies for depressive symptoms among adolescents. These findings may benefit school administrators, teachers, and students. First, we provide school administrators with research evidence on improving the school climate for students’ mental health that can be used to develop and implement school climate improvement actions. Based on our research findings, school administrators should strive to prevent a negative school climate. This can be achieved by initiating campaigns to promote civility among students and implementing policies that address and correct inappropriate behavior among teachers. Second, our study provides school administrators and teachers with new ideas for promoting students’ psychological well-being by enhancing students’ school belonging. Specifically, this study suggests that school administrators and teachers should establish information exchange platforms for students so that they can report any pressures and difficulties, enabling the school to take measures to help students solve their problems. Additionally, schools could organize rich campus cultural activities to stimulate students’ sense of collective honor, and provide lectures on various adolescent issues to enhance adolescents’ understanding of their own growth and promote mental health. It is important to note that our findings and suggestions are based solely on observational studies, which is a study with a low level of evidence according to the guidelines of evidence-based medicine (Kunnamo, 2005). The prevention of and interventions for depressive symptoms among adolescents involve a multitude of stakeholders, including government entities, schools, communities, families, and individuals, and is a rather complex process. This also underscores the need for psychologists to further explore the mechanisms behind adolescent depressive symptoms and potential intervention strategies from a broader perspective. This exploration is crucial to establish a solid foundation for a comprehensive system for preventing and managing depressive symptoms in the future.

This study contributes to the literature in several ways. First, it used a nationally representative, large-sample survey, which allowed for good extrapolation of the findings. Second, it used a longitudinal study design, and the associations and mediating effects explored suggest directional relationships compared to a cross-sectional study. Third, it included a range of covariates in its analysis of the mediating role of school belonging, thereby excluding confounding biases and rendering the results more robust.

There are also a few limitations to the study. First, although the longitudinal design used in this study allowed for the exploration of the longitudinal associations between negative school climate, school belonging, and depressive symptoms, the causal relationship between the three remains unclear. Second, the surveys used self-reporting instruments, which are subject to social desirability response bias. In particular, with regard to the negative school climate as reported by school administrators, the administrators may have reduced the frequency of negative incident reports in order to present a favorable impression. Third, the limitations associated with the measurement of school climate and school belonging should not be overlooked. The measurements might not fully capture the concepts and connotations of these variables due to the limitations inherent in the secondary data used. Future research should consider employing more comprehensive measurement tools and conducting rigorous psychometric analyses.

## 5 Conclusion

Among Chinese adolescents, a negative school climate was associated with a greater risk of depressive symptoms; however, school belonging mediated this association. In other words, school belonging explained the relationship between negative school climate and depressive symptoms. The results of this study contribute to a deeper understanding of the psychological mechanism contributing to depressive symptoms in adolescents from the perspective of school climate. This study suggests that improving school belonging may be helpful in decreasing the impact of a negative school climate on depressive symptoms in adolescents.

## Data availability statement

The datasets presented in this article are not readily available because the author does not have the authority to disclose the data of this study. Requests to access the datasets should be directed to the China Education Panel Survey project team ceps@nsrcruc.org.

## Ethics statement

The studies involving humans were approved by The Ethics Committee of Renmin University of China approved the implementation of the CEPS. The studies were conducted in accordance with the local legislation and institutional requirements. Written informed consent was obtained from the students, schoolteachers, and parents/guardians of the students before the baseline survey.

## Author contributions

YY: Writing – original draft. QS: Writing – review & editing. SL: Writing – review & editing.

## References

[ref1] Akaltun İ. Çayır A. Kara T. Ayaydın H. (2018). Is growth hormone deficiency associated with anxiety disorder and depressive symptoms in children and adolescents?: a case-control study. Growth Hormon. IGF Res. 41, 23–27. doi: 10.1016/j.ghir.2018.06.001 29886327

[ref2] Bonell C. P. Fletcher A. Jamal F. Wells H. Harden A. Murphy S.. (2013). Theories of how the school environment impacts on student health: systematic review and synthesis. Health Place 24, 242–249. doi: 10.1016/j.healthplace.2013.09.014, PMID: 24177419

[ref3] Cole D. A. Maxwell S. E. (2003). Testing mediational models with longitudinal data: questions and tips in the use of structural equation modeling. J. Abnorm. Psychol. 112, 558–577. doi: 10.1037/0021-843X.112.4.558, PMID: 14674869

[ref4] Gámez-Guadix M. (2014). Depressive symptoms and problematic internet use among adolescents: analysis of the longitudinal relationships from the cognitive–behavioral model. Cyberpsychol. Behav. Soc. Netw. 17, 714–719. doi: 10.1089/cyber.2014.0226, PMID: 25405784 PMC4238256

[ref5] Goodenow C. (1993). The psychological sense of school membership among adolescents: scale development and educational correlates. Psychol. Sch. 30, 79–90. doi: 10.1002/1520-6807(199301)30:1<79::AID-PITS2310300113>3.0.CO;2-X

[ref6] Guwen Z. (2023). Research on the influence of school environment on Students' social and emotional skills. Shanghai: East China Normal University.

[ref7] Hatchel T. Espelage D. L. Huang Y. (2018). Sexual harassment victimization, school belonging, and depressive symptoms among LGBTQ adolescents: temporal insights. Am. J. Orthopsychiatry 88, 422–430. doi: 10.1037/ort0000279, PMID: 28617003

[ref8] Hogg M. A. (2016). Social identity theory. Cham: Springer.

[ref9] Hornsey M. J. (2008). Social identity theory and self-categorization theory: a historical review. Soc. Personal. Psychol. Compass 2, 204–222. doi: 10.1111/j.1751-9004.2007.00066.x

[ref10] Ivie E. J. Pettitt A. Moses L. J. Allen N. B. (2020). A meta-analysis of the association between adolescent social media use and depressive symptoms. J. Affect. Disord. 275, 165–174. doi: 10.1016/j.jad.2020.06.014, PMID: 32734903

[ref11] Jia Y. Way N. Ling G. Yoshikawa H. Chen X. Hughes D.. (2009). The influence of student perceptions of school climate on socioemotional and academic adjustment: a comparison of Chinese and American adolescents. Child Dev. 80, 1514–1530. doi: 10.1111/j.1467-8624.2009.01348.x, PMID: 19765015

[ref12] Jiang Y. Yang F. (2023). Effect of household toilet accessibility on physical health of ethnic minority adolescents: a longitudinal study from the China education panel survey 2013 and 2014. BMC Public Health 23:685. doi: 10.1186/s12889-023-15547-5, PMID: 37046235 PMC10091831

[ref13] Jie L. Lei Z. (2022). The composition of peer's hukou, identification and educational achievement of migrant students. China Econ. Educ. Rev. 7, 84–99. doi: 10.19512/j.cnki.issn2096-2088.2022.05.004

[ref14] Joiner T. E. Timmons K. A. (2010). “Depression in its interpersonal context,” in Handbook of depression. G. I. H. H. C. L. New York: Guilford Press.

[ref15] Klik K. A. Cárdenas D. Reynolds K. J. (2023). School climate, school identification and student outcomes: a longitudinal investigation of student well-being. Br. J. Educ. Psychol. 93, 806–824. doi: 10.1111/bjep.12597 37068920

[ref16] Kline R. B. (2023). Principles and practice of structural equation modeling. Guilford publications.

[ref17] Kunnamo I. (2005). Evidence-based medicine guidelines. Chichester: John Wiley & Sons.

[ref18] Lee T. S.-H. Wu Y.-J. Chao E. Chang C.-W. Hwang K.-S. Wu W.-C. (2021). Resilience as a mediator of interpersonal relationships and depressive symptoms amongst 10th to 12th grade students. J. Affect. Disord. 278, 107–113. doi: 10.1016/j.jad.2020.09.033, PMID: 32956959

[ref19] Lei Y. Yousheng D. (2019). Will family social capital and school environment affect adolescent mental health? Empirical analysis based on CEPS data. China Youth Study 1, 47–56. doi: 10.19633/j.cnki.11-2579/d.2019.0007

[ref20] Li C. Jiang S. (2018). Social exclusion, sense of school belonging and mental health of migrant children in China: a structural equation modeling analysis. Child Youth Serv. Rev. 89, 6–12. doi: 10.1016/j.childyouth.2018.04.017

[ref21] Long E. Zucca C. Sweeting H. (2021). School climate, peer relationships, and adolescent mental health: a social ecological perspective. Youth Soc. 53, 1400–1415. doi: 10.1177/0044118x20970232, PMID: 34848899 PMC7612050

[ref22] Nie Q. Yang C. Teng Z. Furlong M. J. Pan Y. Guo C.. (2020). Longitudinal association between school climate and depressive symptoms: the mediating role of psychological suzhi. Sch. Psychol. 35, 267–276. doi: 10.1037/spq0000374, PMID: 32673054

[ref23] Parr E. J. Shochet I. M. Cockshaw W. D. Kelly R. L. (2020). General belonging is a key predictor of adolescent depressive symptoms and partially mediates school belonging. Sch. Ment. Heal. 12, 626–637. doi: 10.1007/s12310-020-09371-0

[ref24] Patel V. Flisher A. J. Hetrick S. McGorry P. (2007). Mental health of young people: a global public-health challenge. Lancet 369, 1302–1313. doi: 10.1016/S0140-6736(07)60368-7 17434406

[ref25] Pozuelo J. R. Desborough L. Stein A. Cipriani A. (2022). Systematic review and meta-analysis: depressive symptoms and risky behaviors among adolescents in low-and middle-income countries. J. Am. Acad. Child Adolesc. Psychiatry 61, 255–276. doi: 10.1016/j.jaac.2021.05.005 34015483

[ref26] Radloff L. S. (1977). The CES-D scale: a self-report depression scale for research in the general population. Appl. Psychol. Meas. 1, 385–401. doi: 10.1177/014662167700100306

[ref27] Renick J. Reich S. M. (2021). Best friends, bad food, and bullying: how students' school perceptions relate to sense of school belonging. J. Community Psychol. 49, 447–467. doi: 10.1002/jcop.22471, PMID: 33225487

[ref28] Rudasill K. M. Snyder K. E. Levinson H. Adelson L. (2018). Systems view of school climate: a theoretical framework for research. Educ. Psychol. Rev. 30, 35–60. doi: 10.1007/s10648-017-9401-y

[ref29] Shorey S. Ng E. D. Wong C. H. J. (2022). Global prevalence of depression and elevated depressive symptoms among adolescents: a systematic review and meta-analysis. Br. J. Clin. Psychol. 61, 287–305. doi: 10.1111/bjc.12333, PMID: 34569066

[ref30] Slaten C. D. Ferguson J. K. Allen K.-A. Brodrick D.-V. Waters L. (2016). School belonging: a review of the history, current trends, and future directions. Educ. Dev. Psychol. 33, 1–15. doi: 10.1017/edp.2016.6

[ref31] Smith W. F. Andrews R. L. (1989). Instructional leadership: How principals make a difference. Alexandria, VA: ERIC.

[ref32] Stirling K. Toumbourou J. W. Rowland B. (2015). Community factors influencing child and adolescent depression: a systematic review and meta-analysis. Aust. N. Z. J. Psychiatry 49, 869–886. doi: 10.1177/0004867415603129, PMID: 26416916

[ref33] Tang X. Tang S. Ren Z. Wong D. F. K. (2020). Psychosocial risk factors associated with depressive symptoms among adolescents in secondary schools in mainland China: a systematic review and meta-analysis. J. Affect. Disord. 263, 155–165. doi: 10.1016/j.jad.2019.11.118 31818773

[ref34] Van Orden K. A. Witte T. K. Cukrowicz K. C. Braithwaite S. R. Selby E. A. Joiner T. E. Jr. (2010). The interpersonal theory of suicide. Psychol. Rev. 117, 575–600. doi: 10.1037/a0018697, PMID: 20438238 PMC3130348

[ref35] Wang M.-T. Degol J. L. (2016). School climate: a review of the construct, measurement, and impact on student outcomes. Educ. Psychol. Rev. 28, 315–352. doi: 10.1007/s10648-015-9319-1

[ref36] Way N. Reddy R. Rhodes J. (2007). Students’ perceptions of school climate during the middle school years: associations with trajectories of psychological and behavioral adjustment. Am. J. Community Psychol. 40, 194–213. doi: 10.1007/s10464-007-9143-y, PMID: 17968655

[ref37] Welsh W. N. (2000). The effects of school climate on school disorder. Ann. Am. Acad. Pol. Soc. Sci. 567, 88–107. doi: 10.1177/000271620056700107

[ref38] WHO. (2021). Mental health of adolescents. Available at: https://www.who.int/news-room/fact-sheets/detail/adolescent-mental-health

[ref39] Wong M. D. Dosanjh K. K. Jackson N. J. Rünger D. Dudovitz R. N. (2021). The longitudinal relationship of school climate with adolescent social and emotional health. BMC Public Health 21:207. doi: 10.1186/s12889-021-10245-6, PMID: 33485308 PMC7825179

[ref40] Zhai B. Li D. Li X. Liu Y. Zhang J. Sun W.. (2020). Perceived school climate and problematic internet use among adolescents: mediating roles of school belonging and depressive symptoms. Addict. Behav. 110:106501. doi: 10.1016/j.addbeh.2020.106501, PMID: 32634681

[ref41] Zheng M. Guo X. Deng J. Hu M. (2023). Association between interpersonal relations and anxiety, depression symptoms, and suicidal ideation among middle school students. Front. Public Health 11:194. doi: 10.3389/fpubh.2023.1053341 PMC9971595 36866094

