# Supplementary material for: School belonging mediates the association between negative school climate and depressive symptoms among Chinese adolescents: a national population-based longitudinal study
Source: Front Psychol. 2024 May 24;15:1368451. doi: 10.3389/fpsyg.2024.1368451 (PMC11160120; doi:10.3389/fpsyg.2024.1368451)
Supplement: Supplementary file 1 [file Table_1.docx]

School belonging mediates the association between negative school climate and depressive symptoms among Chinese adolescents: A national population-based longitudinal study

Supplementary materials

Supplementary Table 1 Descriptive analysis of school administrators

| Variables |  |
| --- | --- |
| Age, mean ± SD | 44.3 ± 5.9 |
| Years of work experience, mean ± SD | 23.7 ± 6.2 |
| Sex, n (%) |  |
| Male | 90 (80.4) |
| Female | 22 (19.6) |
| Position |  |
| Principal | 60 (53.6) |
| Vice principal | 39 (34.8) |
| Director of education | 10 (8.9) |
| Others | 3 (2.7) |
| Educational level, n (%) |  |
| Junior college education | 13 (11.6) |
| Undergraduate degree | 90 (80.4) |
| Graduate degree | 9 (8.0) |
